# Supplementary material for: Patterns of Intron Gain and Loss in Fungi
Source: PLoS Biol. 2004 Nov 30;2(12):e422. doi: 10.1371/journal.pbio.0020422 (PMC532390; doi:10.1371/journal.pbio.0020422)
Supplement: Table S1 — Also available at http://genes.mit.edu/NielsenEtAl/. (4.3 MB ZIP). [file pbio.0020422.st001.zip › NielsenEtAl/html/516.html]

AN4087.1.NCU00489.1.MG05673.1.FG05433.1


```
 CLUSTAL W (1.82) Multiple Sequence Alignments - Introns Inserted


Sequence 1: NCU00489.1	263 aa
Sequence 2: MG05673.1	258 aa
Sequence 3: FG05433.1	255 aa
Sequence 4: AN4087.1	264 aa
Alignment Length: 267 aa
Number Identitical Residues: 217 aa
Alignment Score (without introns) 8184


MG05673.1 	MLP~S----SSKRRKFVADGVFYAELNEFFQRELAEEGYSGVEVRVTPTVTDIII~RATH
NCU00489.1	MTA~SLSVYSSKRRKFVADGVFYAELNEFFQRELAEEGYSGVEVRVTPTVTDIII~RATH
FG05433.1 	MRI2S------KRRKFVADGVFYAELNEFFQRELAEEGYSGVEVRVTPTVTDIII~RATH
AN4087.1  	MAA~VQG-AISKRRKFVADGVFYAELNEFFQRELAEEGYSGVEVRVTPTVTDIST2--TH
          	*     .   :******************************************     **

MG05673.1 	TQEVLGEQGRRIRELTSLIQKRFKFPADSVSLYAAKVQNRGLSAVAQCESLRYKLLNGLA
NCU00489.1	TQEVLGEQGRRIRELTSLIQKRFKFPENSVSLYAAKVQNRGLSAVAQCESLRYKLLNGLA
FG05433.1 	TQEVLGEQGRRIRELTSLIQKRFKFPENSVSLYAAKVQNRGLSAVAQCESLRYKLLNGLA
AN4087.1  	TQEVLGEQGRRIRELTSLIQKRFKFPENSVSLYAAKVQNRGLSAVAQCESLRYKLLNGLA
          	************************** :********************************

MG05673.1 	VRRACYGVLRFIMESGAKGCEVVVSGKLRAARAKSMKFT~DGFMIHSGQPAKDFIDSATR
NCU00489.1	VRRACYGVLRFIMESGAKGCEVVVSGKLRAARAKSMKFT~DGFMIHSGQPAKDFIDSATR
FG05433.1 	VRRACYGVLRFIMESGAKGCEVVVSGKLRAARAKSMKFT~DGFMIHSGQPAKDFIDHATR
AN4087.1  	VRRACYGVLRFIMESGAKGCEVVVSGKLRAARAKSMKFT0DGFMIHSGQPAKEFIDSATR
          	*************************************** ************:*** ***

MG05673.1 	HVLLRQGVLGIKVKIMRGSDPEGKAGPQKSLPDAVTILEPKEEQPVVQPVSQDYGAKAAQ
NCU00489.1	HVLLRQGVLGIKVKIMRGSDPEGKSGPQKSLPDAVTIIEPKEEQPVTQPISQDYGAKAAQ
FG05433.1 	HVLLRQGVLGIKVKIMRGSDPEGKAGPQKTLPDAVTIIEPKEEQAVLQPVSQDYGAKAAQ
AN4087.1  	HVLLRQGVLGIKVKIMRGSDPEGKAGPQKTLPDSVTIIEPKEEQPVLQPISQDYGAKAIA
          	************************:****:***:***:******.* **:********  

MG05673.1 	VQAAAEAARQAEQ-GEGEEQEPAAEQ----
NCU00489.1	VQAAAEAARQEEQAGEEEAAAPAAEE----
FG05433.1 	AAQAAQDARVAEE--EGGEEVQAVEQ----
AN4087.1  	AQQAAEQQRLAEQQAAEGQEGGAAETYAQE
          	.  **:  *  *: .       *.*  :..
```
